# *Bifidobacterium lactis* BL-99 modulates intestinal inflammation and functions in zebrafish models

**DOI:** 10.1371/journal.pone.0262942

**Published:** 2022-02-16

**Authors:** Meng Chen, Chinfeng Liu, Mingzhu Dai, Qinwen Wang, Chunqi Li, Weilian Hung

**Affiliations:** 1 Inner Mongolia Dairy Technology Research Institute Co. Ltd., Hohhot, China; 2 Yili Innovation Center, Inner Mongolia Yili Industrial Group Co., Ltd., Hohhot, China; 3 Hunter Biotechnology, Inc., F1A, Hangzhou, China; Jiangsu University, CHINA

## Abstract

This study was designed to explore the therapeutics and the mechanisms of a patented and marked gastric acid and intestine juice-resistant probiotics *Bifidobacterium lactis* BL-99 (*B*. *lactis* BL-99) on the intestinal inflammation and functions in the zebrafish models. After feeding for 6 hours, *B*. *lactis* BL-99 was fully retained in the larval zebrafish intestinal tract and stayed for over 24 hours. *B*. *lactis* BL-99 promoted the intestinal motility and effectively alleviated aluminum sulfate-induced larval zebrafish constipation (*p* < 0.01). Irregular high glucose diet induced adult zebrafish intestinal functional and metabolic disorders. After fed with *B*. *lactis* BL-99, *IL-1β* gene expression was significantly down-regulated, and *IL-10* and *IL-12* gene levels were markedly up-regulated in this model (*p* < 0.05). The intestinal lipase activity was elevated in the adult zebrafish intestinal functional disorder model after *B*. *lactis* BL-99 treatment (*p* < 0.05), but tryptase content had no statistical changes (*p* > 0.05). *B*. *lactis* BL-99 improved the histopathology of the adult zebrafish intestinal inflammation, increased the goblet cell numbers, and up-and-down metabolites were markedly recovered after treatment of *B*. *lactis* BL-99 (*p* < 0.05). These results suggest that *B*. *lactis* BL-99 could relieve intestinal inflammation and promote intestinal functions, at least in part, through modulating intestinal and microbial metabolism to maintain intestinal health.

## Introduction

Probiotics consumption for health promotion and well-being has increased worldwide in recent years [1] and various types of foods have been supplemented with probiotics [2]. Probiotics have also been commercialized in the form of lyophilized powder [3]. Probiotics are live beneficial microorganisms ingested into the gastrointestinal tract with food or water, regulating health by affecting internal microbial to achieve a balanced state [4, 5]. In aquaculture, probiotics and prebiotics play an important role and provide health benefits in improving growth performances, disease resistance, immunity, intestinal immune barrier integrity, intestinal microbiota, and water quality [6–8].

The most studied probiotic candidates in aquaculture belong to the Firmicutes phylum, namely lactic acid-producing bacteria (LAB) and *Bacillus spp* [5, 9–13]. Although they are poorly adapted to and/or rarely uncommon in marine environment, LAB can tolerate acidic pH and bile salts properties, allowing them to survive in the intestinal systems [14, 15]. Studies have proven that there are several types of probiotic strains that can be effectively used in aquafeeds with unique and beneficial properties, such as *Bifidobacterium*, *Lactobacillus*, *Bacillus*, and several other bacterial species [16–18]. These probiotics can survive and colonize in the intestinal mucus, help the processing and uptake of feed, and promote the growth of the fish [19, 20]. LAB bacteria have been isolated from the intestines of salmonids [21], and some of these strains tested for their antibacterial effect and ability to inhibit adhesion of *Aeromonas hydrophila*, *A*. *salmonicida*, *Yersinia ruckeri*, and *V*. *anguillarum* to intestinal mucus from rainbow trout (*in vitro*) [10]. Dietary probiotic supplementation can prevent zebrafish intestinal microbiota dysbiosis and lipid metabolism disorders after exposure to perfluorobutane sulfonate (PFBS) [22], and dietary supplementation for the probiotic *L*. *rhamnosus* also counteracts zebrafish neurotoxicity caused by PFBS [23].

Inability to acid and gastrointestinal juice is a common property of *Bifidobacterium*, which makes it difficult to reach and colonize in the intestine through gastric juice [24]. *Bifidobacterium lactis* BL-99 (*Bifidobacterium animalis* subsp. *lactis* BL-99, *B*. *lactis* BL-99) was originally isolated from the intestines of a Chinese healthy infant [25, 26] and patented and marketed by Inner Mongolia Yili Industrial Group [27, 28]. This probiotics strain was resistant to gastric acid and intestinal juice and the live bacteria were more than 61% in pH 2.5 gastric acid solution and 70% in pH 6.8 small intestine juice after treatment for 2 hours (hrs) [27].

*B*. *lactis* BL-99 has no exogenous antibiotic resistance genes [27] and has passed the determination of bacterial resistance to meet the requirements of the European Food Safety Authority (EFSA) for the evaluation of drug resistance of edible bacteria. This strain was found to be negative for mucin degradation and platelet aggregation and had no genetic mutagenicity. *B*. *lactis* BL-99 was confirmed no dose-dependent mortality and toxicity throughout multidose oral toxicity tests in mice and rats and thus generally recognized as safe (GRAS) status as a probiotic [29]. *In vivo* experiments in mice showed that *B*. *lactis* BL-99 significantly promoted the growth of intestinal *Bifidobacteria* and Lactic acid bacteria, and inhibited *Desulfovibrio* and/or *Enterobacter*, especially Helicobacter pylori and/or Escherichia-Shiga Bacteria [27, 30]. This strain increased the phagocytic rate and phagocytic index of mouse macrophages in the carbon clearance experiment and elevated the number of antibody-producing cells in sheep erythrocytes (SRBC) immunized mice [28].

The research of probiotics on intestinal microbial balance, intestinal functions, inflammation, and intestinal metabolites, etc. mostly use traditional mammalian models. Conventional mammalian enteritis models are chemical-induced, for example, DSS (3,6-Disinapoylsucrose) was used to induce mice colitis [31], and TNBS (2,4,6-trinitro-Benzenesulfonic acid) was applied to induce guinea pig colitis [32]. It is necessary to fast for 24–36 hrs before chemical drug induction to allow the animals to empty their feces, this is not consistent with the intestinal environment of patients with enteritis and cannot completely simulate the patient’s conditions. Very recently, there were a few papers on mammals that mimicked the intestinal harm caused by irregular diet, high sugar and fat [33–35], and these experimental periods were 8–12 weeks long with high costs. There is an urgent need to establish an efficient and rapid animal model system to investigate probiotics.

Zebrafish (*Danio rerio*) intestinal composition is similar to that of humans, e.g., connective tissue, external-longitudinal muscle and circular muscle, et al. [36, 37]. With the implementation of the “3R principle (Reduction, Replacement, Refinement)”, zebrafish has been used as an alternative model organism to screen intestinal beneficial bacteria [38, 39], but there are relatively few evaluations of the intestinal vitality and functions of probiotics [37, 40], and lack of in-depth research on the efficacies and mechanisms of new and novel probiotic strains in the zebrafish models. In this study, we therefore assessed the effects of *B*. *lactis* BL-99 on the digestive enzymes, motility, inflammation and metabolites in the larval and adult zebrafish models.

## Materials and methods

### Zebrafish husbandry

Wild-type AB strain at 5 days post fertilization (5 dpf) and adult male zebrafish at 3.5 months post fertilization (3.5 mpf) were used in this study. Zebrafish were maintained at 28°C in fish water (0.2% Instant Ocean Salt in deionized water, pH 6.9–7.2, conductivity 480–510 μS/cm and hardness 53.7–71.6 mg/L CaCO3). The zebrafish facility and the laboratory at Hunter Biotechnology, Inc. are accredited by the Association for Assessment and Accreditation of Laboratory Animal Care (AAALAC) International [41, 42], by the China National Accreditation Service for Conformity Assessment (CNAS), and by China Inspection Body and Laboratory Mandatory Approval (CMA). After each experiment, all the zebrafish were anesthetized and euthanized with 0.25 g/L tricaine methanesulfonate [43], which conforms to the American Veterinary Medical Association (AVMA) requirements for euthanasia by anesthetic [44]. This study was approved by the Institutional Animal Care and Use Committee (IACUC) at Hunter Biotechnology, Inc. and the IACUC approval number was 001458.

### Probiotic strain and culture conditions

*B*. *lactis* BL-99 was deposited in the China Common Microbial Culture Collection and Management Center (CGMCC 15650) on April 26, 2018 [29] and identified by 16S rRNA gene amplified using the universal primers 27F (5’-AGA GTT TGA TCC TGG CTC AG-3’) and 1492R (5’-GGT TAC CTT GTT ACG ACT T T-3’) [45]. The standard *B*. *lactis* BL-99 culture was proliferated with De Man Rogosa Sharpe (MRS) medium (Solarbio, Beijing) supplemented with 0.05% (w/v) L-cysteine (MRSC) for 12–48 hrs at 37°C aerobically [26] and the anaerobic environment was obtained with Anaero Gen sachets (Oxoid Ltd., West Heidelberg/Vic., Australia). Colony-forming unit (CFU) of *B*. *lactis* BL-99 was 1.5*10^11^ CFU / g and preserved at -80°C.

### Chemicals and reagents

Tricaine methanesulfonate (cat. # 886-86-2) and aluminum sulfate (cat. # D1909026) were ordered from Shanghai Aladdin Bio-Chem Technology Co., Ltd (Shanghai, China), nile red (cat. # MKBP6198V) from Sigma-Aldrich (St.Louis, MO, USA), and glucose (lot. # 20201105) was purchased from Sinopharm Chemical Reagent Co., Ltd (Shanghai, China). Tissue cell fixation solution at 4% concentration (cat. # AR-0211-250 mL) was ordered from Beijing Dingguo Changsheng Biotechnology Co., Ltd (www.dingguo.com). CM-DiI cell-labeling solution (CM-DiI, cat. # 2123588) and trizol reagent (cat. # 12183555) were bought from Thermo Fisher Scientific (China) Co., Ltd. FastKing RT Kit (With gDNase) (cat. # KR116-02) was bought from TIANGEN BioTec (Beijing) Co., Ltd (www.tiangen.com), and iTaq Universal SYBR(R) Green Supermix was purchased from BIO-RAD Co., Ltd. (www.bio-rad.com). Fish trypsin ELISA kit (item no. ml064285) was bought from Shanghai Enzyme-linked Biotechnology Co., Ltd. (Shanghai, China) (www.mlbio.cn). Lipase (LPS) kit (item no. A054-2-1) was bought from Nanjing Jiancheng Bioengineering Institute (Nanjing, China) (www.njjcbio.com).

### *B*. *lactis* BL-99 labeling with fluorescent dye

After collection, *B*. *lactis* BL-99 were fluorescently labeled by incubating with 10 μg/ml CM-DiI (chloromethyl benzamide derivatives of 1,1’-dioctadecyl-3,3,3’,3’-tetramethylindo-carbocyanine perchlorate, Molecular Probes, Eugene, OR) containing 0.5% DMSO in PBS [46] at 37°C for 20 min. After washing in PBS for 3 times, the labeled *B*. *lactis* BL-99 were treated with larval zebrafish for its retaining time determination in the intestinal tract and for its effects on the intestinal motility and digestion functions as described below. The dye is transferred from mother to daughter bacteria and fluorescent *B*. *lactis* BL-99 were clearly visible in the zebrafish intestinal tract.

### Intestinal retaining time and lasting period determination of *B*. *lactis* BL-99 in larval zebrafish

Wild-type larval zebrafish at 5 dpf were distributed into 6-well microplates (Nest Biotech, China), 30 zebrafish per well in 3 ml fish water and treated with fluorescent *B*. *lactis* BL-99 at a density of 2.42*10^8^ CFU/mL at 28°C. The zebrafish intestinal fluorescent images were taken periodically at the designated time points to determine the retaining time of this probiotics. After treatment of fluorescent *B*. *lactis* BL-99 for 24 hrs, the zebrafish were transferred into fish water for 4 and 24 hrs, respectively, 10 zebrafish were randomly selected from each group and at each time point for visual observation and image acquisition under a fluorescent stereomicroscope (AZ100, Nikon, Japan), installed with a high-speed video camera (JVC, Japan). Quantitative image analyses were performed using image-based analysis (NIS-Elements D3.20; Japan), the retaining time and lasting period of *B*. *lactis* BL-99 in the larval zebrafish intestine tract were calculated based on the fluorescent intensity. To protect fluorescent *B*. *lactis* BL-99 from light-induced decomposition, experiments were carried out at a constant temperature (28°C) in the dark. All experiments were performed in duplicate and repeated for at least 3 times.

### Assessing therapeutic effects of *B*. *lactis* BL-99 on the larval zebrafish constipation

The larval zebrafish of AB strain at 5 dpf were distributed into a 6-well microplate, 30 zebrafish per well in 3 ml fish water. The zebrafish constipation model was established by treatment with 1 μg/mL aluminum sulfate [47] at 28°C for 6 hrs, and 50 ng/mL nile red (intestinal chromogenic agent, [48]) was added into the treatment solution for the last 3 hrs. After removing aluminum sulfate and nile red, the zebrafish were continuously treated with *B*. *lactis* BL-99 at concentrations of 2.42*10^6^, 2.42*10^7^ and 2.42*10^8^ CFU/mL, respectively, for 24 hrs. Domperidone was used as a positive control drug. The zebrafish treated with aluminum sulfate and nile red only served as a model control. The zebrafish without any treatment were used as a negative control. At the end of treatments, the zebrafish were imaged under a AZ100 fluorescent stereomicroscope, installed with a high-speed video camera. The therapeutic effects of *B*. *lactis* BL-99 on the larval zebrafish constipation were determined based on the intestinal fluorescent quantitative analyses.

### Adult zebrafish intestinal function disorder model

Seventy-five male adult zebrafish of 3.5 mpf (months post fertilization) wild-type AB strain were transferred into 5 L beaker in a volume of 4 L containing 15 zebrafish. In the initial tests, three concentrations (2.42*10^6^, 2.42*10^7^ and 2.42*10^8^ CFU/mL) were used for *B*. *lactis* BL-99 treatment. Untreated control zebrafish were examined in parallel. The adult zebrafish were housed in a light and temperature-controlled aquaculture facility with a standard 14:10 h light/dark photoperiod. (1) Days 1–3 of the experiment: except for the untreated control zebrafish, the resting groups were not fed and starved for 3 days. *B*. *lactis* BL-99 groups were treated with this probiotic at 3 designated concentrations, respectively, as described above in fish water every day during the daytime for 8 hrs and then lived in fresh fish water; (2) Days 4–17: *B*. *lactis* BL-99 groups were treated with this probiotic during the daytime for 8 hrs and then transferred into 3% glucose in fish water for 16 hrs. The model zebrafish were only treated with 3% glucose for 16 hrs and the untreated control zebrafish were fed with brine shrimp twice a day. On the 18th day of the experiment, the zebrafish intestinal tissues were collected and the intestinal digestive enzymes, inflammatory and immunity factor genes and histopathology were examined, respectively, and the interventional effects of *B*. *lactis* BL-99 were assessed.

### Inflammation and immune genes analyses

To explore the possible anti-inflammation and the intestinal immune mechanisms of *B*. *lactis* BL-99, the mRNA levels of interleukin-1β (*IL-1β*), interleukin-10 (*IL-10*) and interleukin-12 (*IL-12*) were determined in the adult zebrafish intestines by real-time quantitative PCR (qPCR) [49]. Briefly, after *B*. *lactis* BL-99 treatment, total RNA was extracted from 10 homogenized zebrafish per group using trizol reagent. About 2 μg total RNA of each sample was used for cDNA synthesis using FastQuant RT Kit (With gDNase) and qPCR amplifications were carried out with a CFX Connect detection system (Bio Rad, Singapore) using the iTaq Universal SYBR Green Supermix in which there were three technical or biological replicates. The qPCR protocol was 2 minutes at 95°C-40 cycles of 5 seconds at 95°C-30 seconds at 60°C. Expression data was normalized against the expression of β-actin and the relative quantification of each gene mRNA among groups was calculated as follows: The relative expression of RNA = 2^ΔΔC(t); ΔΔC(t) = ΔC(t)_Model_—ΔC(t)_Probiotics_; ΔC(t) = ΔC(t)_Target gene_—ΔC(t)_β-actin._ The primers used in this study were as follows: *β-ACTIN*-FOR: TCGAGCAGGAGATGGGAACC, *β-ACTIN*-REV: CTCGTGGATACCGCAAGATTC (GenBank accession numbers 57934) [49, 50], *IL-1β*-FOR: GTCACACTGAGAGCCGGAAG, *IL-1β*-REV GCAGGCCAGGTACAGGTTAC (interleukin 1 beta, GenBank accession numbers 405770) [49], *IL-10*-FOR: TTCAGGAACTCAAGCGGGAT, *IL-10*-REV: AAGAGCAAATCAAGCTCCCCC (interleukin 10, GenBank accession numbers 553957) [49], *IL-12*-FOR: AACTCCTACAAGCCCAGCAC, *IL-12*-REV: ACACTCGGTCGTCAAACGAA (interleukin 12a, GenBank accession numbers 445410). Each primer pair was designed using NCBI/Primer-BLAST.

### Digestive enzyme assays

In order to evaluate the effects of *B*. *lactis* BL-99 on the intestinal functions of the adult zebrafish, ELISA kits were used to determine the intestinal tissue lipase activity and trypsin content. The optical density (OD) values were measured by multifunctional microplate reader (SPARK, TECAN, Switzerland) at wavelength 595 nm for the protein concentration, 580 nm for the lipase activity, and 450 nm for the trypsin content, respectively. The lipase activity and trypsin content per gram of protein in zebrafish intestinal tissues were calculated based on the OD values.

### Intestinal histopathology

To confirm the intestinal damage caused by the irregular high-sugar diet, and the effects of *B*. *lactis* BL-99 intervention, we performed the gut histopathological examinations on the adult zebrafish. At the end of the experiments, zebrafish intestinal tissues were fixed in 4% paraformaldehyde in 0.1 M phosphate buffered saline for 4 hrs at 4°C, dehydrated in graded series of ethanol solutions before paraffin embedding. Embedded zebrafish intestines were longitudinally sectioned at 5 μm and stained with hematoxylin and eosin (H&E) [41, 51]. Thirty zebrafish were used for each group. Histological images were obtained using a histological microscope (CX31RTSF, Olympus, Japan) with a digital camera (TS 2000, Sony, Japan), and pathological diagnosis was completed by a certified pathologist.

### Metabolomics analyses

Ten adult zebrafish whole guts from each group were used for the intestinal metabolite extraction and the metabolomic analysis. Twenty-five mg intestinal tissues from each gut were homogenized with 800 μL pre-cold precipitation agent (methanol: acetonitrile: pure water = 2:2:1, v/v). After sonication on ice for 10 minutes, let the mixture stand at -20°C for 120 minutes, followed by centrifugation at 25000 g for 15 min at 4°C. Six hundred μL of supernatant was taken and put in a freeze-drying machine to drain and reconstituted in 600 μL of 10% methanol solution. After ultrasound and centrifugation, the supernatant was chromatographed using 2777C UPLC system (Waters, UK), and the eluted small molecules were collected in positive and negative ion modes using Xevo G2-XS QTOF (Waters, UK). Metabolite resonances were identified according to the information from the Human Metabolome Database (HMDB) and Kyoto Encyclopedia of Genes and Genomes (KEGG). Significantly changed metabolites between the control and treatment groups were identified following the criteria below: *p* < 0.05 and fold change ≥ 1.2 or fold change ≤ 0.8333 and VIP ≥ 1; and statistically significant changes in at least two dose groups. Student’s t test was used for the statistical analyses of the metabolites.

### Statistical analyses

One-way ANOVA followed by the Dunnett’s test was used to compare differences among groups. All statistical analyses were performed using the GraphPad software (GraphPad Prism, version 5.0, USA), and *p* < 0.05, *p* < 0.01 and *p* < 0.001 were all considered statistically significant. For quantitative analysis, all data were presented as mean ± SEM, and results were statistically compared between the probiotics-treated and model zebrafish groups. All experiments were repeated for at least 3 times. Zebrafish natural death in untreated groups was ≤ 10%, and all intra- and inter-group coefficient of variation (CV) was ≤ 25%.

## Results

### Studies in the larval zebrafish

#### The retaining and lasting time periods of *B*. *lactis* BL-99

**Fig 1:**
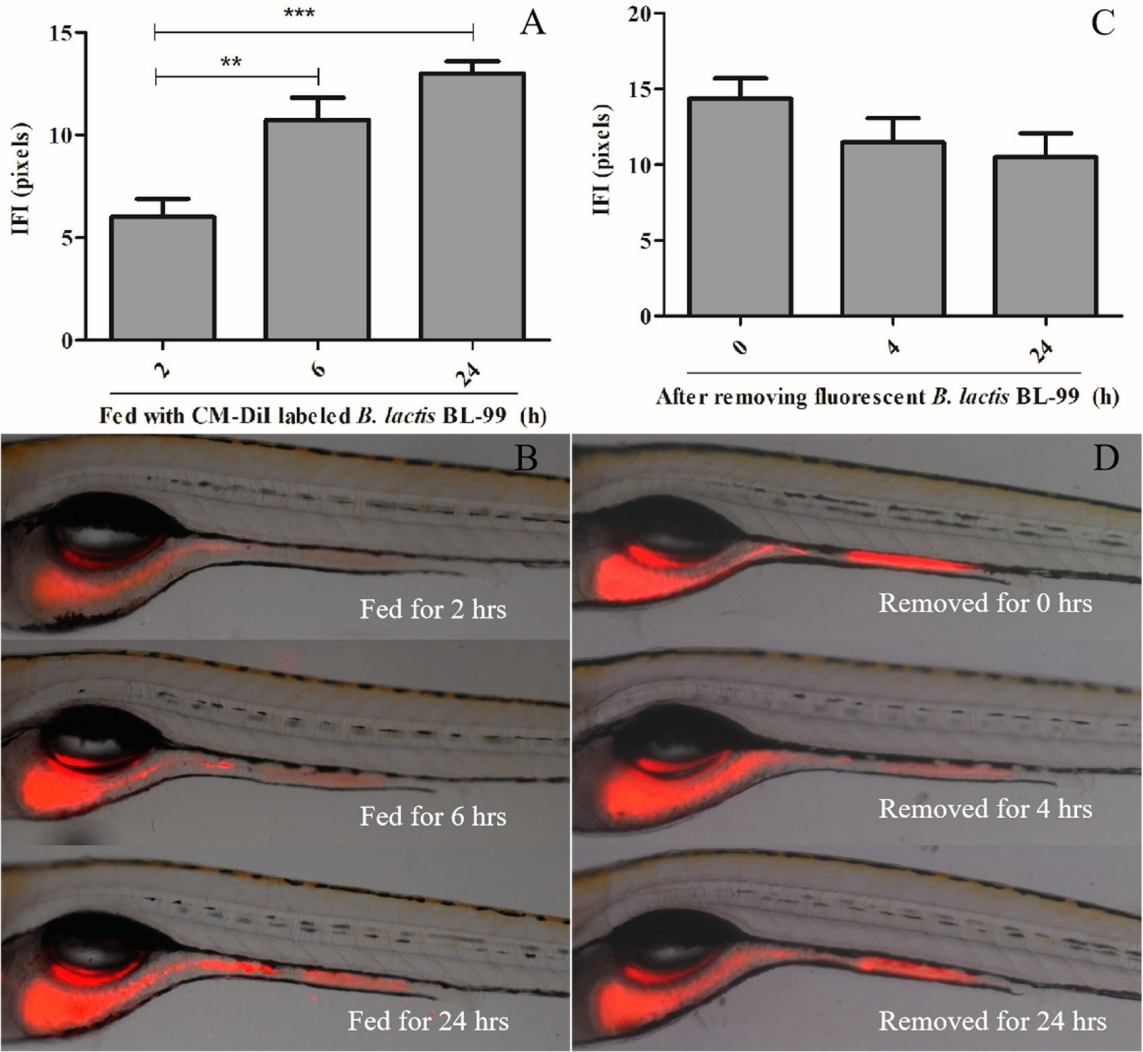
The retaining and lasting time periods of *B*. *lactis* BL-99 in the larval zebrafish intestinal tracts. The larval zebrafish were fed with CM-DiI labeled *B*. *lactis* BL-99 for 2, 6 and 24 hrs, the fluorescent intensities (A) and quantitative analyses (B) in the larval zebrafish intestinal tracts. After removing fluorescent *B*. *lactis* BL-99 from the treatment solutions and transferred the zebrafish to fresh fish water for 0, 4 and 24 hrs, the larval zebrafish intestinal fluorescence (C) and quantitative analyses (D). Data were expressed as means ± S.E.M. Compared with the model group, **p < 0.01, ***p < 0.001. IFI = intestinal fluorescent intensity.

As indicated in Fig 1A and 1B, after fed with CM-DiI labeled *B*. *lactis* BL-99 for 2, 6 and 24 hrs, the fluorescent intensities in the larval zebrafish intestinal tracts were 6.02± 0.866, 10.7± 1.08 and 13.0 ± 0.601 pixels, respectively. Comparing the fluorescent intensities between 24 hr and 2 hr feeding, *p* < 0.001, but *p* > 0.05 when comparing the fluorescent intensities between 24 hr and 6 hr feeding, suggesting that *B*. *lactis* BL-99 effectively retained in the larval zebrafish intestinal tract after 6 hr feeding.

After removing fluorescent *B*. *lactis* BL-99 from the treatment solutions and transferred the zebrafish into fresh fish water for 0, 4 and 24 hrs, the larval zebrafish intestinal fluorescence was 14.4± 1.31, 11.5± 1.58 and 10.5± 1.57 pixels (Fig 1C and 1D), and no any statistically significant differences among the groups, implying that *B*. *lactis* BL-99 could last in the larval zebrafish intestines for over 24 hrs.

#### The therapeutic effects on the intestinal motility and constipation

**Fig 2:**
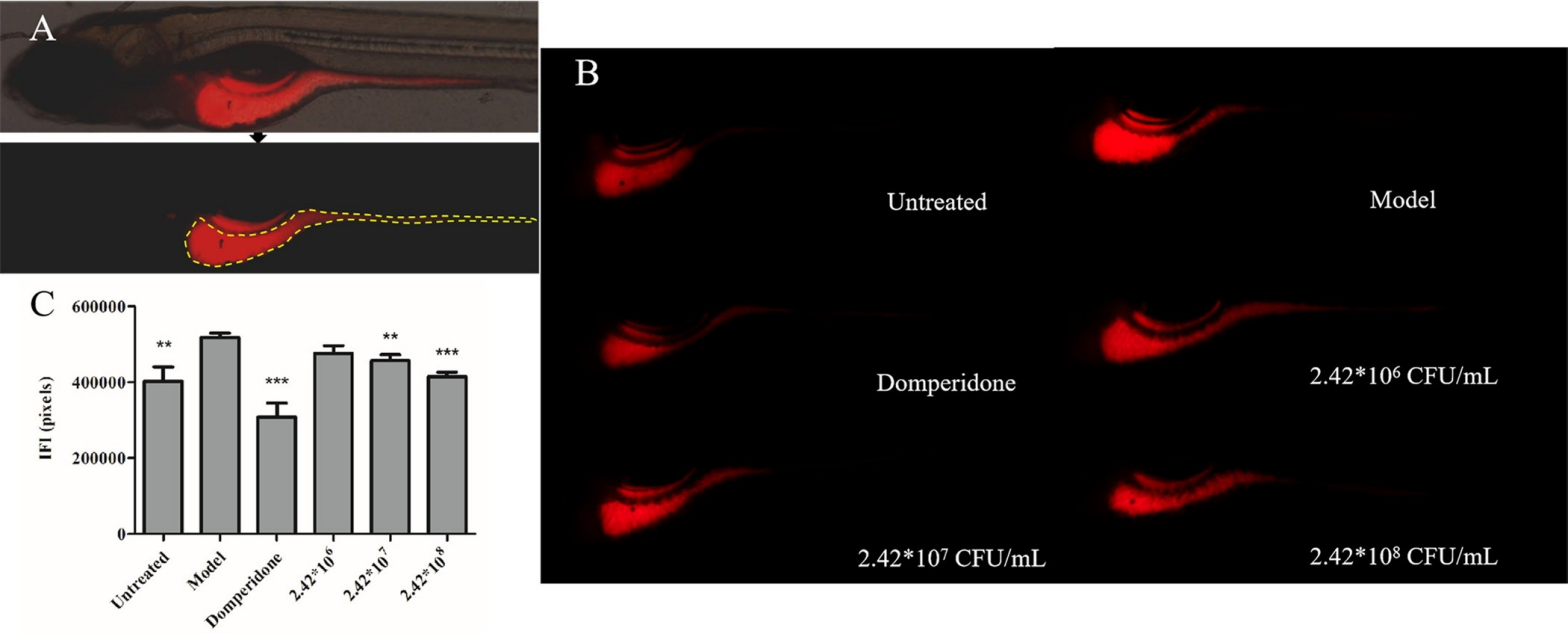
The therapeutic effects of *B*. *lactis* BL-99 on the larval zebrafish intestinal motility and constipation. Schematic diagram of the intestinal fluorescent *B*. *lactis* BL-99 and analysis area of the larval zebrafish (A). The nile red fluorescent intensity (B) and quantitative analyses (C) in the larval zebrafish intestines. Data were expressed as means ± S.E.M. Compared with the model group, **p < 0.01, ***p < 0.001. IFI = intestinal fluorescent intensity.

As demonstrated in Fig 2B and 2C, the nile red fluorescent intensity in the normal larval zebrafish intestines was 403493 ± 37456 pixels, and 517757 ± 11985 pixels in the aluminum sulfate-treated zebrafish (*p* < 0.01), indicating that the larval zebrafish constipation model was successfully established. The positive control drug Domperidone significantly promoted the intestinal motility (fluorescent pixels = 308784 ± 36464, *p* < 0.001 as compared with the constipation model zebrafish). The dose-dependent intestinal fluorescent intensity decreases (476071 ± 20633, 456847 ± 15814 and 414652 ± 11561 pixels) were found in the constipation zebrafish treated with *B*. *lactis* BL-99 at 2.42*10^6^ (476071 ± 20633 pixels), 2.42*10^7^ (456847 ± 15814 pixels) and 2.42*10^8^ CFU/mL (414652 ± 11561 pixels), respectively (*p* > 0.05, *p* < 0.01, *p* < 0.001).

### Studies in the adult zebrafish

#### Inflammation and immune gene expression

**Fig 3:**
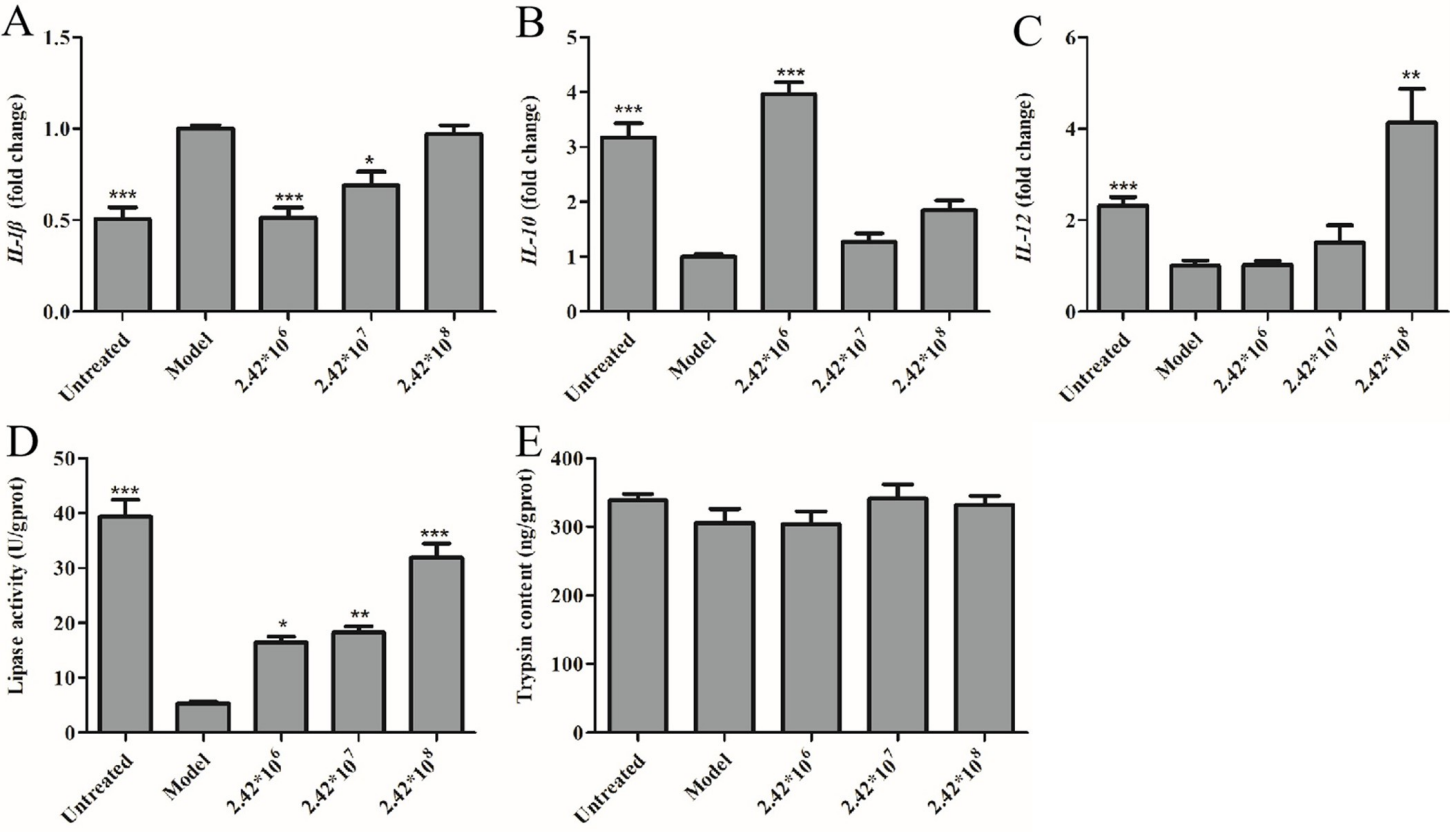
The inflammatory and immunity gene expression and the digestive enzyme quantifications in the intestinal tract tissues of the irregularly high-glucose diet-induced intestinal functional disorders of adult zebrafish. ***IL****-1β* gene levels (A), *IL-10* gene levels (B) and *IL-12* gene levels (C) in the adult zebrafish intestines. Lipase activity (D) and trypsin content (E) in the adult zebrafish intestines. Data were expressed as means ± S.E.M. Compared with the model group, *p < 0.05, **p < 0.01, ***p < 0.001.

The purity of the extracted RNA (A260/A280) was in the range of 1.95–2.12. As shown in Fig 3A, 3% glucose-treated zebrafish showed an upregulation of the *IL-1β* gene expression. A concentration-dependent downregulations of the *IL-1β* gene expression was observed in the model zebrafish treated with 2.42*10^6^, 2.42*10^7^ and 2.42*10^8^ CFU/mL of *B*. *lactis* BL-99, and the decreases were 0.511± 0.055, 0.691± 0.072 and 0.969± 0.049 folds, respectively, relative to the model group (*p* < 0.001, *p* < 0.05, *p* > 0.05).

As demonstrated in Fig 3B, 3% glucose-treated zebrafish showed a downregulation of the *IL-10* gene expression. After treatment with *B*. *lactis* BL-99 at the concentrations of 2.42*10^6^, 2.42*10^7^ and 2.42*10^8^ CFU/mL, *IL-10* and *IL-12* expression levels were elevated to 3.96± 0.219, 1.27± 0.150 and 1.85± 0.176 folds (*p* < 0.001, *p* > 0.05, *p* > 0.05) and 1.01± 0.097, 1.51± 0.368 and 4.13± 0.745 folds (*p* > 0.05, *p* < 0.01, *p* < 0.001), respectively, relative to the model group (Fig 3C).

#### Lipase activity and trypsin content

As shown in Fig 3D, 3% glucose-treated zebrafish showed a reduction of the intestinal lipase activity. A concentration-dependent augmentation of the lipase activity was observed in the model zebrafish treated with 2.42*10^6^, 2.42*10^7^ and 2.42*10^8^ CFU/mL of *B*. *lactis* BL-99, and the lipase activity were 16.4± 1.10, 18.2± 1.15 and 31.8± 2.58 U/g protein, respectively, relative to the model group (*p* < 0.05, *p* < 0.01, *p* < 0.001). *B*. *lactis* BL-99 had no statistically significant effect on the intestinal trypsin content, although it showed an increased trend as indicated in Fig 3E.

#### Intestinal histopathology

**Fig 4:**
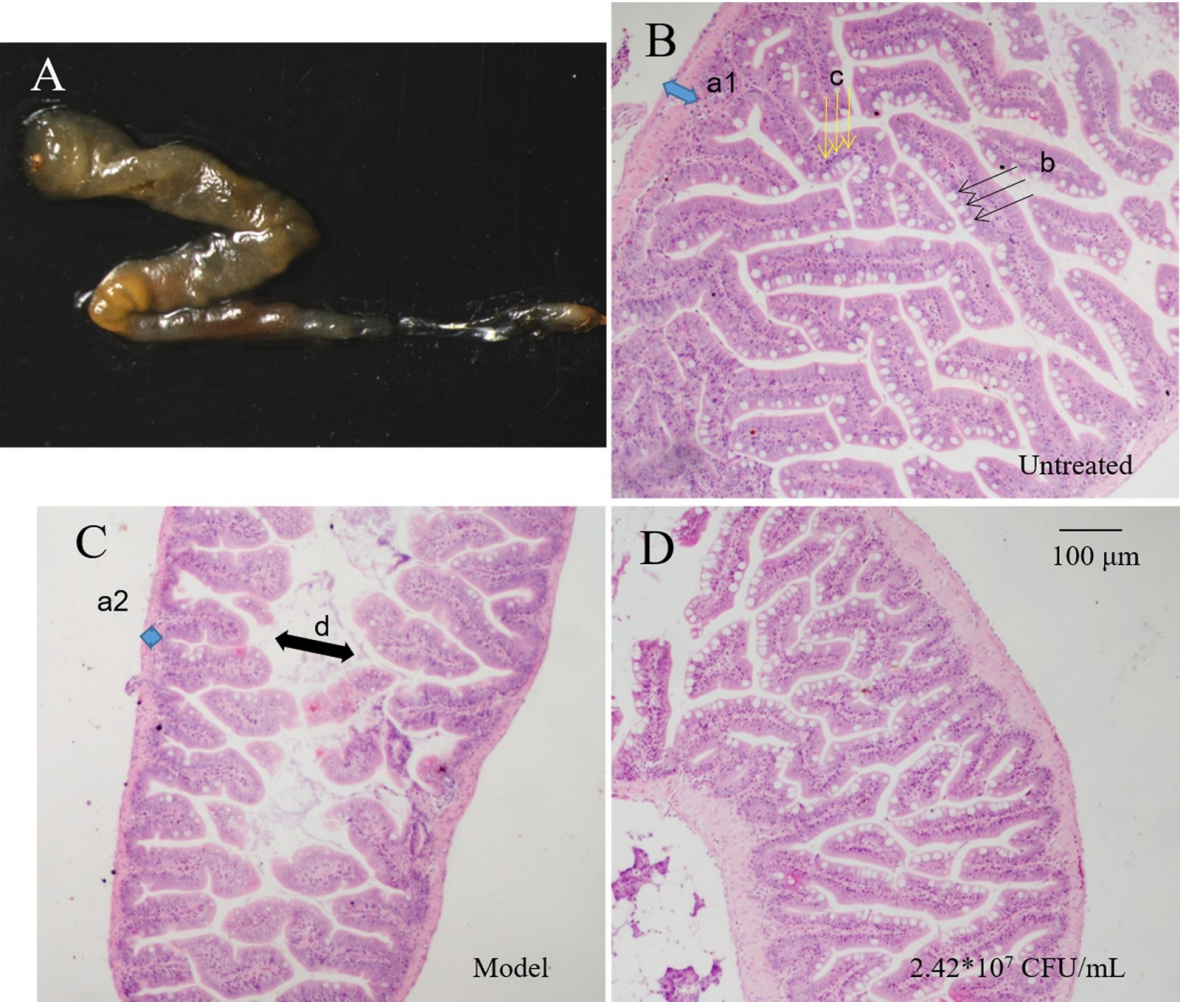
Histopathology of the irregularly high glucose diet-induced intestinal functional disorders in adult zebrafish intestines treated with *B*. *lactis* BL-99. A fresh complete intestine of normal adult zebrafish (A). Normal (untreated) zebrafish intestinal H & E staining (B). The intestinal functional disorder zebrafish (model) intestinal histopathology (C). The intestinal functional disorder zebrafish treated with *B*. *lactis* BL-99 at 2.42*10^7^ CFU/mL (D). Muscle layer (a1, blue double-headed arrow); goblet cells (b, black one-way arrow); lymphocytes (c, yellow one-way arrow); thinner intestinal wall (a2, blue square) and dilation of the intestinal lumen (d, black double-headed arrow).

At the end of the experiment, a freshly complete intestine of normal (untreated) adult zebrafish was taken and shown in Fig 4A. The subsequent H&E staining demonstrated that the gut of normal zebrafish had thicker intestinal walls, well-developed muscle layer (a1, blue double-headed arrow) and intestinal villi. The intestinal villi were high in height, large in area, and staggered branched or finger-shaped. Normal intestinal goblet cells (b, black one-way arrow) were numerous, with large and round heads, arranged in rows between intestinal villi epithelial cells; lymphocytes (c, yellow one-way arrow) were distributed in a monolayer of columnar epithelium inside the cell (Fig 4B). In the model group, the zebrafish intestinal wall became thinner (a2), the villi were sparse and the height was significantly reduced, the intestinal cavity was dilated (d, black double arrow); the number of goblet cells were significantly reduced; the number of lymphocytes were decreased (Fig 4C). Compared with the model group, the *B*. *lactis* BL-99 treatment at 2.42*10^7^ CFU/mL led to the developed high villi, increased goblet cells and columnar epithelial cells, and the gut tissue morphology was closely similar to that of normal zebrafish (Fig 4D).

#### Metabolic characteristics

**Fig 5:**
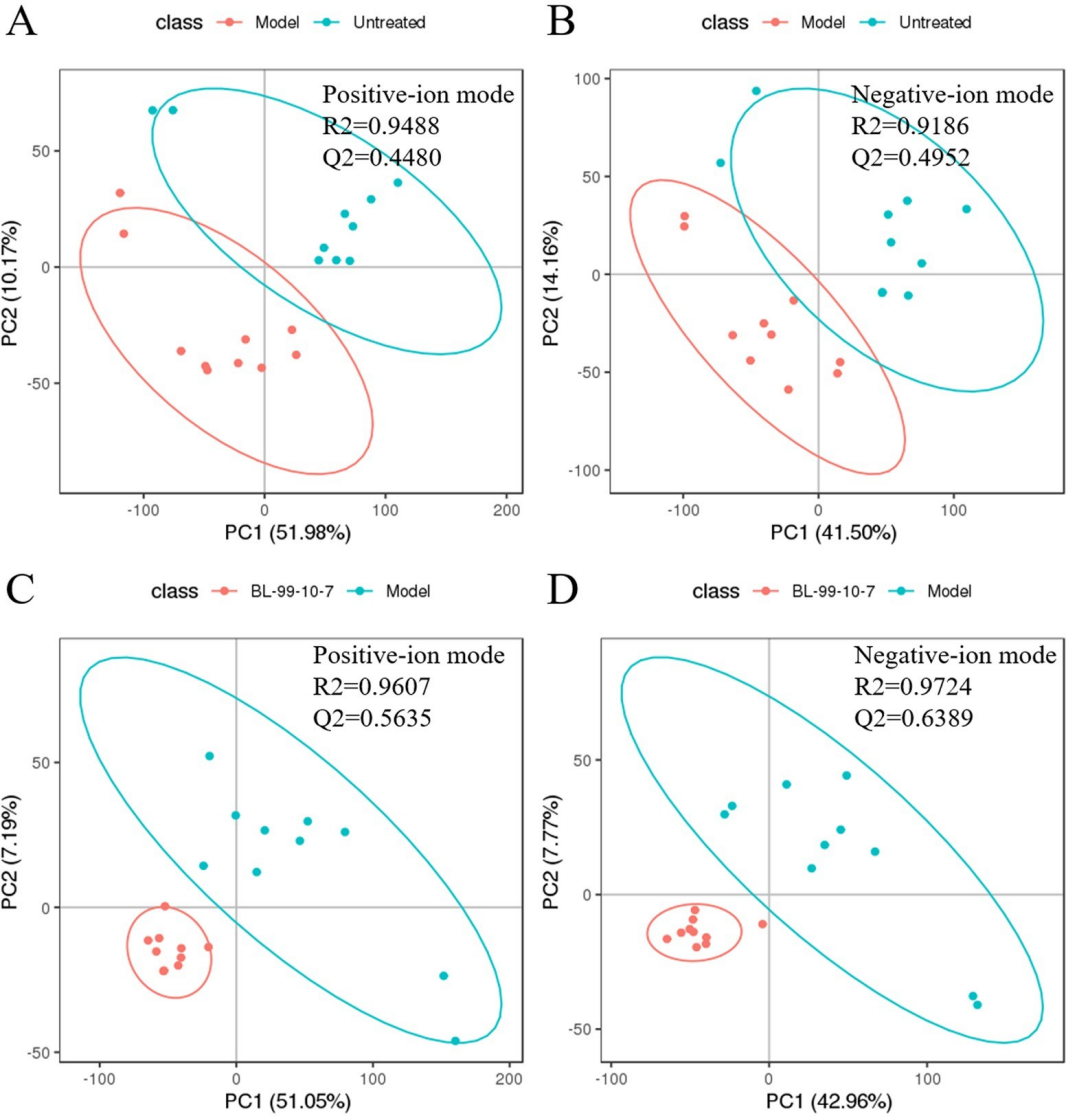
Partial least-squares discriminant analysis (PLS-DA) for the intestinal metabolic profiles. (A) was in the positive ion mode and (B) in negative ion mode between the intestinal functional disorder adult zebrafish (model) and normal (untreated); and (C) was in the positive ion mode and (D) in negative ion mode between the intestinal functional disorder adult zebrafish (model) and the model zebrafish treated with *B*. *lactis* BL-99 at 2.42*10^7^ CFU/mL (BL-99-10-7). These ellipses represented the 95% confidence region.

As shown in Fig 5, in the positive and negative ion modes, the normal control group and the model group showed significant separation (pos:5A, neg:5B), the *B*. *lactis* BL-99 with concentration of 2.42*10^7^ CFU/mL (BL-99-10-7) and the model group showed complete separation (pos:5C, neg:5D), and the degrees of aggregations among the BL-99-10-7 treatment groups were obvious. There were 106 positive-ion metabolites and 218 negative-ion metabolites were statistically significantly changed in the intestines as compared between normal and the model zebrafish; and 213 positive-ion metabolites and 402 negative-ion metabolites with significant differences between the model zebrafish and the model zebrafish treated with *B*. *lactis* BL-99.

**Fig 6:**
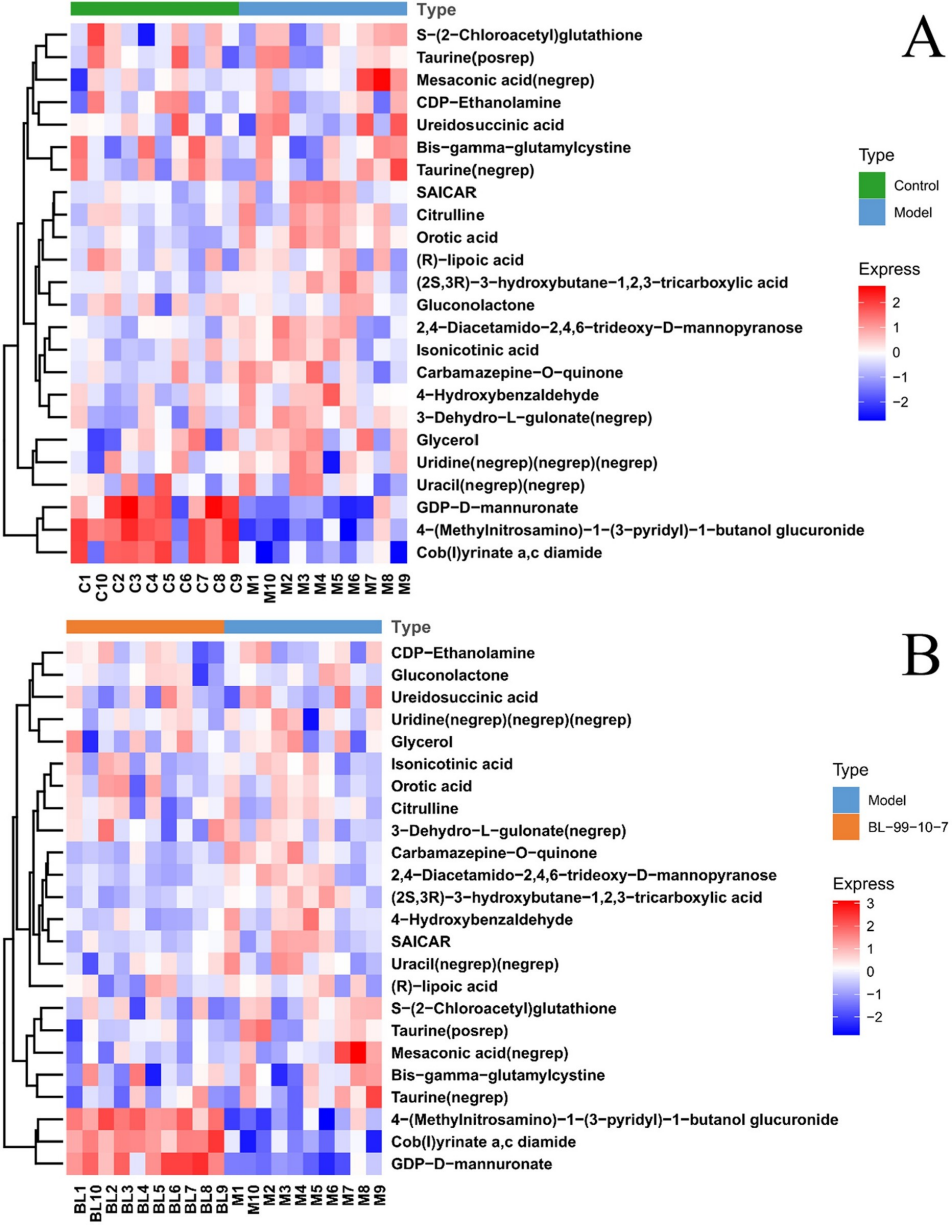
Heat map of significantly changed metabolites. Statistically markedly changed metabolites between the intestinal functional disorder adult zebrafish (model) and normal control (untreated) zebrafish (A); and between the model zebrafish treated with and without *B*. *lactis* BL-99 (B).

Heat maps of significantly changed metabolites between the intestinal functional disorder zebrafish (model) and normal (untreated) zebrafish were indicated in Fig 6A, and between the model zebrafish treated with and without *B*. *lactis* BL-99 (6B). As shown in Tables 1 and 2, 20 metabolites were increased and 3 were decreased in the intestinal functional disorder zebrafish when compared with normal zebrafish, and theses up-and-down metabolites were recovered after treatment of *B*. *lactis* BL-99. Among the 23 significantly different intestinal metabolites, 13 metabolites were identified with the known physiological and pathological functions: citrulline, glycerol, CDP-Ethanolamine, gluconolactone, uridine, uracil, taurine, mesaconic acid, ureidosuccinic acid, orotic acid, 4-hydroxybenzaldehyde, bis-γ-glutamylcystine and R-lipoic acid. The biological significances for the remaining 10 metabolites below were not known or unclear yet: SAICAR, isonicotinic acid, GDP-d-mannuronate, 3-dehydro-L-gulonate, (2S,3R)-3-hydroxybutane-1,2,3-tricarboxylic acid, cob (I) yrinate a,c diamide, 4-(Methylnitrosamino)-1-(3-pyridyl)-1-butanol glucuronide, s-(2-chloroacetyl)glutathione, 2,4-diacetamido-2,4,6-trideoxy-d-mannopyranose, and carbamazepine-o-quinone.

**Table 1:** Negative-ion metabolites in the intestinal function disorder zebrafish after *B*. *lactis* BL-99 treatment.

KEGG.ID	RT(min)	M/Z	Ratio	t. test	Result		Metabolite	Pathway
C00198	0.75	223.046	0.223	0.024	down	(Untreated/Model)	Gluconolactone	map00030
			0.163	0.006	down	(BL-99-10-7/Model)		
C00116	1.89	73.029	0.281	0.002	down	(Untreated/Model)	Glycerol	map00561
			0.415	0.019	down	(BL-99-10-7/Model)		
C00618	0.69	193.035	0.064	0.000	down	(Untreated/Model)	Dehydro-L-gulonate	map00040
			0.305	0.040	down	(BL-99-10-7/Model)		
C00245	4.13	124.007	0.366	0.016	down	(Untreated/Model)	Taurine	map00430
			0.285	0.001	down	(BL-99-10-7/Model)		
C00327	0.68	174.088	0.247	0.001	down	(Untreated/Model)	Citrulline	map00220
			0.343	0.012	down	(BL-99-10-7/Model)		
C04823	1.01	435.055	0.065	0.002	down	(Untreated/Model)	SAICAR	map00230
			0.085	0.007	down	(BL-99-10-7/Model)		
C00438	5.98	221.042	0.363	0.020	down	(Untreated/Model)	Ureidosuccinic acid	map00240
			0.179	0.003	down	(BL-99-10-7/Model)		
C00106	3.87	111.019	0.153	0.042	down	(Untreated/Model)	Uracil	
			0.043	0.006	down	(BL-99-10-7/Model)		
C00295	1.09	155.009	0.053	0.000	down	(Untreated/Model)	Orotic acid	
			0.356	0.036	down	(BL-99-10-7/Model)		
C00299	4.07	225.052	0.123	0.010	down	(Untreated/Model)	Uridine	
			0.096	0.026	down	(BL-99-10-7/Model)		
C01732	3.96	129.019	0.143	0.001	down	(Untreated/Model)	Mesaconic acid	map00630
			0.130	0.000	down	(BL-99-10-7/Model)		
C04593	0.78	205.035	0.183	0.001	down	(Untreated/Model)	(2S,3R)-3-hydroxybutane-1,2,3-tricarboxylic acid	map00640
			0.150	0.001	down	(BL-99-10-7/Model)		
C16241	3.69	205.036	0.416	0.012	down	(Untreated/Model)	-lipoic acid	map00785
			0.385	0.007	down	(BL-99-10-7/Model)		
C06505	5.45	935.358	5.167	0.017	up	(Untreated/Model)	Cob (I) yrinate a,c diamide	map00860
			3.719	0.008	up	(BL-99-10-7/Model)		
C19605	0.6	366.129	2.883	0.000	up	(Untreated/Model)	4-(Methylnitrosamino)-1-(3-pyridyl)-1-butanol glucuronide	map00980
			2.306	0.001	up	(BL-99-10-7/Model)		
C14864	2.11	382.045	0.653	0.025	down	(Untreated/Model)	(2-Chloroacetyl) glutathione	
			0.258	0.001	down	(BL-99-10-7/Model)		
C00633	5.47	121.029	0.149	0.000	down	(Untreated/Model)	Hydroxybenzaldehyde	map01100
			0.173	0.001	down	(BL-99-10-7/Model)		

**Table 2:** Positive-ion metabolites in the intestinal function disorder zebrafish after *B*. *lactis* BL-99 treatment.

KEGG.ID	RT(min)	M/Z	Ratio	t. test	Result		Metabolite	Pathway
C20424	1.58	285.084	0.117	0.001	down	(Untreated:Model)	2,4-Diacetamido-2,4,6-trideoxy-D-mannopyranose	map00520
			0.089	0.001	down	(BL-99-10-7/Model)		
C07446	1.32	124.039	0.149	0.001	down	(Untreated/Model)	Isonicotinic acid	map00983
			0.229	0.011	down	(BL-99-10-7/Model)		
C16606	4.03	267.074	0.280	0.004	down	(Untreated/Model)	Carbamazepine-O-quinone	map00982
			0.124	0.000	down	(BL-99-10-7/Model)		
C03646	8.26	499.112	0.296	0.029	down	(Untreated/Model)	Bis-gamma-glutamylcystine	map00480
			0.193	0.006	down	(BL-99-10-7/Model)		
C00570	3.52	429.059	0.462	0.026	down	(Untreated/Model)	CDP-Ethanolamine	map00564
			0.177	0.008	down	(BL-99-10-7/Model)		
C00245	0.62	148.004	0.679	0.050	down	(Untreated/Model)	Taurine	map00120
			0.106	0.000	down	(BL-99-10-7/Model)		
C00976	6.14	658.015	6.580	0.004	up	(Untreated/Model)	GDP-D-mannuronate	map00051
			9.432	0.000	up	(BL-99-10-7/Model)		

## Discussion

*B*. *lactis* has been confirmed as a gastric acid and intestinal juice tolerable probiotics [21] that makes it favorable to reach and colonize in the intestine. In this investigation, we found that *B*. *lactis* BL-99 effectively preserved in the larval zebrafish intestinal tract after 6 hrs of feeding and stayed in the intestinal tract for over 24 hrs. *B*. *lactis* BL-99 promoted the intestinal motility and relieved the constipation in aluminum sulfate-induced larval zebrafish model. This patented and marked probiotics increased digestive enzyme lipase production, regulated inflammatory and immune responses, and relieved intestinal inflammation in an irregularly high-glucose diet-induced adult zebrafish intestinal functional disorder model. These findings imply that *B*. *lactis* BL-99 could be an effective and probably potent modulator of the intestinal functions for both physiological and pathological conditions.

Orally administered probiotics encounter various challenges on their journey through the mouth, stomach and intestinal tract. The health benefits of probiotics are diminished mainly due to the substantial reduction of viable probiotic bacteria under the harsh conditions in the gastrointestinal cavity and the colonization resistance caused by commensal bacteria [52]. In a previous study aimed to evaluate the colonization ability of *L*. *casei* SY13 and explore its effects on gut microbial structure and diversity in mice, the authors found that the stable colonization of *L*. *casei* SY13 was associated with dosage and treatment days, and thus laid a foundation for studying interactions between *L*. *casei* SY13 and other members of the gut microbiota [53]. The long-lasting retention period in the intestinal tract is necessary for *B*. *lactis* BL-99 to play its functions in the intestinal health and the disease prevention and treatment.

In normal digestion, food is transited through the gastrointestinal tract by rhythmic contractions called peristalsis. Slow gastrointestinal contractions could lead to digestive function disorders and constipation [48] that are highly prevalent in any population worldwide [54]. Probiotics have been now commonly used to treat functional gastrointestinal motility disorders with largely varied efficacies [55]. Here we found that *B*. *lactis* BL-99 promoted the intestinal motility and relief constipation and increased the digestive enzyme lipase production in the larval and adult zebrafish models, supporting the uses of this probiotics in preventing and treating dyspepsia and motility disorders.

Sugar consumption has dramatically increased in the past few decades [56] and overconsumption of sugar is closely linked to gut permeability and metabolic diseases [57]. The high-glucose- or high-fructose-fed mice lost gut microbial diversity, characterized by a lower proportion of bacteroidetes and a markedly increased proportion of proteobacteria; increased gut permeability due to alterations to the tight junction proteins caused by gut inflammation [33]. In this study, an irregular 3% glucose diet was given to the adult zebrafish for 2 weeks, and the intestinal inflammation and functional disorders were induced as revealed by the elevated intestinal inflammatory factor *IL-1β* gene expression, reduced intestinal immune factors *IL-10* and *IL-12* gene levels, lessened intestinal lipase activity, damaged intestinal histology, and disordered intestinal metabolomics. After *B*. *lactis* BL-99 treatment, the adult zebrafish intestinal inflammation was alleviated, the intestinal immune responses were enhanced, and the intestinal mucus barrier and histopathology were ameliorated.

**Fig 7:**
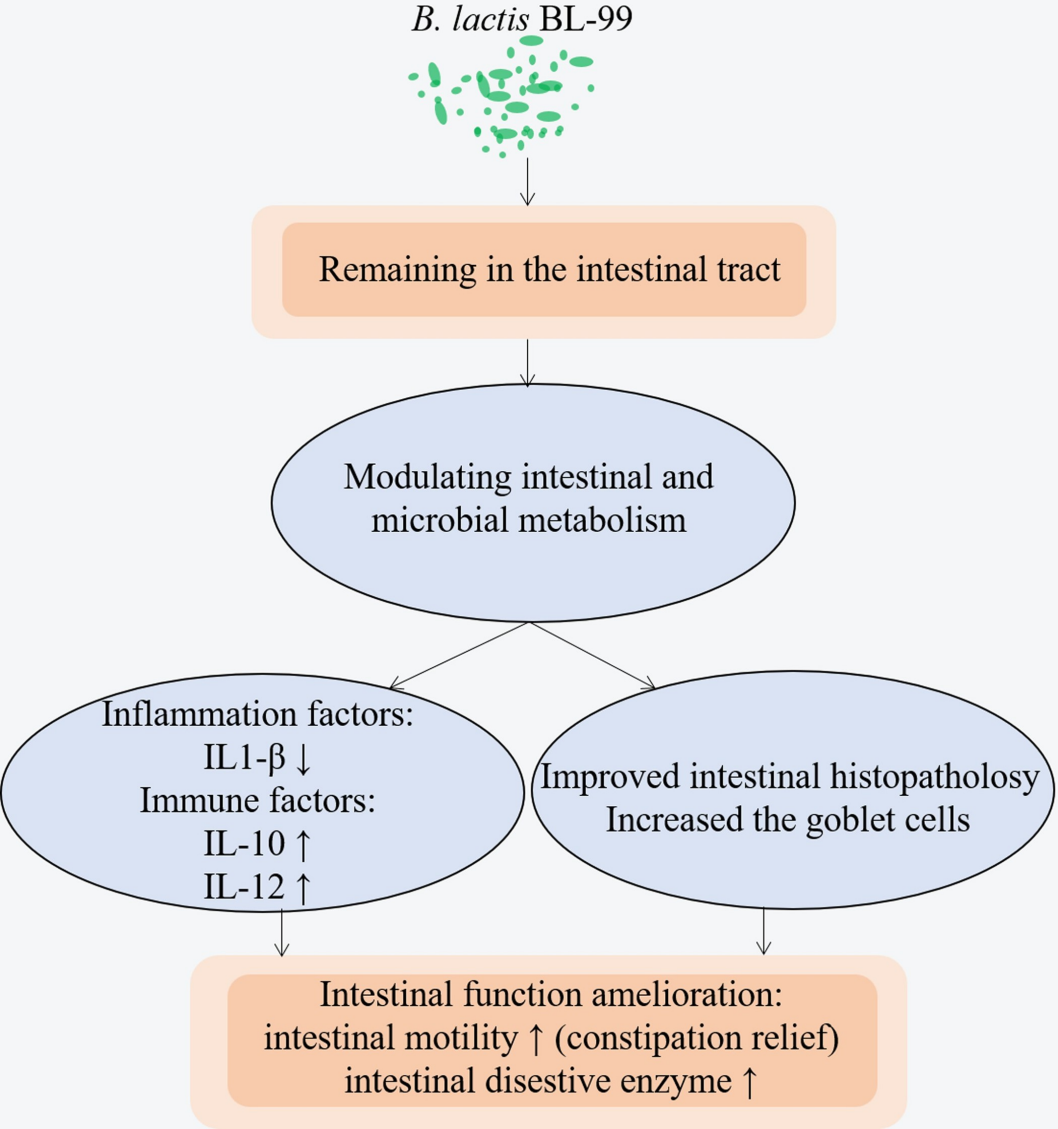
Possible mechanisms of *B*. *lactis* BL-99 modulated the intestinal inflammation and functions.

Interestingly, the gut metabolic disorders, including the intestinal cell and intestinal microbiota metabolism, were observed in the 3% glucose-induced adult zebrafish intestinal function disorder model. For instance, 6 intestinal cell function-related metabolites (citrulline, glycerol, CDP-ethanolamine, gluconolactone, uridine and uracil) and 5 intestinal microbiota-related metabolites (taurine, mesaconic acid, ureidosuccinic acid, orotic acid and 4-hydroxybenzaldehyde) were found statistically different in the intestines between the high-glucose fed and untreated control zebrafish. These 11 metabolites, plus 2 organic compounds bis-γ-glutamylcystine and R-lipoic acid, were all significantly increased in the gut of 3% glucose-fed zebrafish. Surprisedly, *B*. *lactis* BL-99 treatment recovered these intestinal and microbiota metabolites to the levels similar or close to the normal control zebrafish. These results suggest that *B*. *lactis* BL-99 could relieve intestinal inflammation and promote intestinal functions, probably at least in part, through modulating intestinal and microbial metabolism to maintain intestinal health (Fig 7). These and other significant metabolites identified in this work as well as the intestinal microbiota will be further investigated on their roles in the therapeutic mechanisms of *B*. *lactis* BL-99 in the future studies.

## Supporting information

S1 File (XLS)
